# Identification of appropriate biochemical parameters and cut points to detect Maturity Onset Diabetes of Young (MODY) in Asian Indians in a clinic setting

**DOI:** 10.1038/s41598-023-37766-x

**Published:** 2023-07-14

**Authors:** Ramasamy Aarthy, Kathryn Aston-Mourney, Anandakumar Amutha, Antonina Mikocka-Walus, Ranjit Mohan Anjana, Ranjit Unnikrishnan, Saravanan Jebarani, Ulagamathesan Venkatesan, Sundaramoorthy Gopi, Venkatesan Radha, Viswanathan Mohan

**Affiliations:** 1 Madras Diabetes Research Foundation (ICMR Centre for Advanced Research on Diabetes), Chennai, India; 2 School of Medicine, IMPACT, Institute for Innovation in Physical and Mental Health and Clinical Translation, Deakin University Geelong, Geelong, Australia; 3 School of Psychology, Deakin University Geelong, Melbourne, Australia; 4 Dr. Mohan’s Diabetes Specialties Centre (IDF Centre of Excellence in Diabetes Care), No 4, Conran Smith Road, Gopalapuram, Chennai, 600086 India

**Keywords:** Endocrinology, Health care, Medical research

## Abstract

Maturity Onset Diabetes of the Young (MODY) is a monogenic form of diabetes which is detected by genetic testing. We looked at clinical and biochemcial variables that could help detect possible MODY among Asian Indians with youth-onset diabetes. From the diabetes electronic medical records of a diabetes care centre in Chennai in southern India, demographic, anthropometric, and biochemical details of 34 genetically confirmed MODY participants were extracted. They were compared with patients with type 1 diabetes (T1D) (n = 1011) and type 2 diabetes (T2D) (n = 1605), diagnosed below 30 years of age. Clinical and biochemical variables including body mass index (BMI), glycated hemoglobin, HDL cholesterol, and C-peptide (fasting and stimulated) were analyzed to determine whether cut points could be derived to identify individuals who could be sent for genetic testing to diagnose or rule out MODY in this ethnic group. The age at diagnosis was higher for T2D (26.5 ± 4.0 years) compared to T1D (18.2 ± 6.1 years) and MODY (17.8 ± 6.0 years). Individuals with MODY had BMI, glycated hemoglobin, total cholesterol, triglycerides, HDL cholesterol, and C-peptide levels which were intermediate between T1D and T2D. The identified probable parameters and their cut points to identify cases for MODY genetic screening were BMI 21.2–22.7 kg/m^2^, glycated hemoglobin 7.2–10%, HDL cholesterol 43–45 mg/dl, fasting C -peptide, 1.2–2.1 ng/ml and stimulated C-peptide, 2.1–4.5 ng/ml. Asian Indians with MODY have clinical features that are intermediate between T1D and T2D and selected biochemical parameters, especially stimulated C peptide cut points were the most useful to diagnose MODY.

## Introduction

In India more than 74 million adults currently have diabetes and the numbers are increasing steadily^1^, with the prevalence of youth onset diabetes also being high^2–4^. While most individuals with youth onset diabetes have type 1 diabetes (T1D), an increasing number of type 2 diabetes (T2D) is being reported. Moreover, other specific types of diabetes such as Maturity Onset Diabetes of the Young (MODY), the commonest monogenic form of diabetes, are also being reported^5^. As early as 1985, a high prevalence of MODY was reported from India^6^. However, as this was based only on the clinical criteria available at the time, as the genes for MODY had not yet been identified, it is likely that this included a significant proportion of younger age onset T2D. Once molecular genetics was developed in the 1990s, several subtypes of MODY were identified^7–11^. However, the contribution of these subtypes to the prevalence of youth onset diabetes and MODY in India remains unknown.

In western populations, MODY accounts for 2–4% of all diabetes in children and young adults and it is a clinically and genetically heterogenous condition^12^. The prevalence of MODY however, varies with geographic location and depends on the criteria used for diagnosis and screening^13^. In the absence of distinctive clinical features, about 80% of individuals with MODY are misdiagnosed as T1D or T2D^14^.

A correct diagnosis has therapeutic and quality of life implications, with certain MODY subtypes e.g., *HNF1A*-MODY3 and *HNF4A*- MODY1 responding better to oral sulfonylureas rather than to metformin or insulin^15^. However, current clinical criteria are neither sensitive nor specific enough to discriminate MODY from T2D or T1D. For an accurate MODY diagnosis, genetic testing is required^14^ but this is often not readily available and is expensive^16^. Due to this, many cases of MODY are missed^17^. Hence it is important to identify the right cases to be referred for genetic testing, especially in low-resource settings like India. Thus, there is a need to identify less expensive but fairly sensitive, clinical and/or biochemical markers to differentiate likely MODY from T1D and T2D^18^.

Some biomarkers, including high-density lipoprotein cholesterol (HDL-C), triglyceride, and C-peptide, have been determined to aid in distinguishing MODY from T1D and T2D^19^. A MODY probability calculator was developed in 2012 for the UK population to estimate the individual’s probability of having MODY^20^. However, these biomarkers and the calculator are less efficient at identifying South Asians with MODY compared to white populations^21^. A similar prediction model has now been developed in China to identify candidates for genetic testing among T1D and T2D^19^.

Some of the challenges faced in the categorization of young diabetes specifically in India, include lower antibody positivity among T1D, relatively high prevalence of fibro-calculous pancreatic diabetes (FCPD), and a lower BMI at diagnosis in young-onset T2D^15^. Therefore, the existing MODY calculators and biomarker approaches have increased limitations in screening Indian populations for MODY^15^.

Identification of simple clinical criteria with optimal cut-off points for the Asian Indian population would be useful to identify possible MODY among those with young onset diabetes and thus could be a valuable tool to decide which participants are to be referred for genetic testing. This paper attempts to identify appropriate clinical and biochemical variables such as body mass index (BMI), glycated hemoglobin (HbA1c), C-peptide (fasting and stimulated), and high-density lipoprotein (HDL)-cholesterol that may be useful to distinguish individuals for MODY genetic screening in southern India.

## Methods and data collection

### Participants

This study is a part of an ongoing program at our centre from 1992 to 2022. The data for the analysis was extracted from the diabetes electronic medical records of our centre which is a large tertiary diabetes care centre in Chennai in south India. All young patients with onset of diabetes below 30 years of age undergo a strict algorithm-based evaluation at the centre. This includes a detailed family history including a pedigree chart, C-peptide assay (fasting and stimulated), GAD and zinc transporter antibodies, serum ketone measurements, an X-ray of the abdomen to rule out FCPD, and clinical examination to look for acanthosis nigricans (as a marker of type 2 diabetes). We also see if they satisfy the modified clinical criteria for MODY suggested by Tattersall and Fajans^22^ which include less than 30 years of age at onset of diabetes, absence of ketosis, family history of diabetes in at least three generations and response to oral hypoglycaemic drugs and if yes, then they are included for genetic testing of MODY.

Thirty-four individuals were identified with genetically confirmed MODY which included *HNF1A*-MODY3 (n = 21, 61.7%), *HNF4A*-MODY1 (n = 10, 29.4%), and *ABCC8*-MODY12 (n = 3, 8.8%) from the 530 participants who underwent genetic screening. A definite genetic diagnosis of MODY was made in these 34 individuals based on gnomAD^23^ and ACMG criteria^24^ for monogenic diabetes testing as described below. To compare the clinical characteristics of MODY and to identify the likely cut-off points for selecting cases to be referred for genetic screening of MODY from a group of individuals with youth onset diabetes, individuals with T1D (n = 1011) and T2D (n = 1605), matched for the duration of diabetes with the MODY groups, were recruited from the diabetes electronic medical records.

### Genetic analysis

Genomic DNA was isolated from the whole blood and direct sequencing of DNA amplified with published primers^25–29^ was carried out using ABI 3500 Genetic Analyzer (Applied Biosystems, Foster City, CA) using the Big Dye terminator V3.1 chemistry. The resulting sequences were compared with the public databases (NCBI: HNF4A-NM_000457, GCK-NM_000162, HNF1A-NM_000545, HNF1B-NM_000458, and ABCC8-NM_000352) and the Genome Aggregation Database (gnomAD) was used to study the functional consequence of the variant^23^. Only those variants which were classified as *pathogenic* or *likely pathogenic* were included^24^.

### Clinical and laboratory measurements

The clinical characteristics included sex, age at diagnosis, and duration of diabetes along with anthropometric indices of height, weight, and body mass index (BMI). For biochemical investigations, venous blood samples were collected after overnight fasting of more than 10 h. For collecting all clinical data, a standardized protocol is followed at the centre and laboratory standardization is also done routinely with national and international agencies. The lab is certified by the National Accreditation Board for Hospitals and Health Care providers (NABH), India. Fasting plasma glucose, serum total cholesterol, serum triglycerides, LDL and HDL cholesterol, blood urea, and serum creatinine were assayed using Beckman Coulter AU2700 (Fullerton, CA) biochemistry analyzer. The glycated hemoglobin (HbA1c) was analyzed by high-performance liquid chromatography using the Variant II Turbo (Bio-Rad, Hercules, CA). C-peptide (fasting and stimulated) was measured using the chemiluminescence method on a Siemens ADVIA Lentaur XPT immunoassay analyzer at first registration at the centre. A standard breakfast of 400 k calories comprising 65% carbohydrate, 20% protein and 15% fat was given, and a post-prandial blood sample was drawn after 90 min for estimation of stimulated C-peptide values. Repeat C-peptide measurements were also done in many patients during follow up. There were no restrictions on their medications on the day of testing as it is not necessary to stop medications^30,31^.

### Ethics

The study was approved by the Institutional Ethics Committees of both participating institutions (Madras Diabetes Research Foundation dated 06 March 2018, Deakin University Australia Ethics Approval number—2019-060). Written informed consent was obtained from all participants aged 18 years or over and assent was obtained from participants less than 18 years of age (in addition to written parental consent). All procedures followed were in accordance with the ethical standards of the responsible committee on human experimentation and with the Helsinki Declaration of 1975, as revised in 2013.

### Definitions

Diabetes was defined when the fasting plasma glucose (FPG) level was ≥ 126 mg/dl (7.0 mmol/l) and/or 2 h post-load glucose level ≥ 200 mg/dl (11.1 mmol/l)^32^ or if there was a self-reported diagnosis of diabetes treated by a physician or if on medications for diabetes.

*T1D* was defined by abrupt symptoms of polyuria, polydipsia, or unexplained weight loss, diabetic ketoacidosis (DKA), presence of glutamic acid decarboxylase (GAD) antibodies, and requirement of insulin from the time of diagnosis for control of hyperglycaemia^30^.

*T2D* was defined by absence of ketosis, absence of pancreatic calculi (on X-ray abdomen), good response to oral hypoglycemic agents for more than two years, and absence of GAD antibodies^30^.

*MODY* was diagnosed based on the presence of a specific mutation on genetic analysis as explained in the earlier section.

### Statistical analysis

Descriptive statistics were provided for continuous and categorical variables. Continuous variables were expressed as mean with standard deviation. Chi-square test was used to compare the categorical variables and were expressed as frequencies and percentages. One-way ANOVA was used to compare groups for continuous variables and *p* < 0.05 was considered statistically significant.

Receiver operating curves (ROC) were plotted using sensitivity and 1- specificity using T1D and T2D as a gold standard. The ROC curves were used to determine the best cut-offs for sensitivity and specificity. The area under the curve (AUC) helped to study the overall performance of the cut-off points. Sensitivity was defined by the proportion of MODY participants with a given risk factor who were identified correctly by the given variable greater or equal to the cut-off point. Specificity was defined as the proportion of MODY participants without the risk factor who were identified below the cut-off point. The point closest to the upper left-hand corner which maximized sensitivity and specificity was selected as the optimal cut point. Positive and negative predictive values and accuracy for MODY predictors for different cut-off points were calculated.

All statistical analyses were conducted using the Statistical Package for the Social Sciences (SPSS, Inc., Windows V 25.0, Chicago).

## Results

The clinical characteristics of participants with MODY (n = 34), T1D (n = 1011), and T2D (n = 1605) are shown in Table 1. While all participants were selected to have an age of diagnosis of diabetes below 30 years, the age of diagnosis was still significantly higher among T2D when compared to T1D and MODY. Fasting plasma glucose, HbA1c, and HDL cholesterol levels were higher among T1D as compared to MODY and T2D. C-peptide (fasting and stimulated) values in MODY were intermediate between those of T1D and T2D.

**Table 1:** Clinical characteristics of MODY compared to Type 1 diabetes(T1D) and Type 2 diabetes(T2D) participants.

Variables	MODY^+^ (n = 34)	T1D (n = 1011)	T2D (n = 1605)
Sex—Male n (%) #	10 (29.4%)	607 (60%)	1075 (67%)
Age at diagnosis (years)	17.8 ± 6.0	18.2 ± 6.1	26.5 ± 4.0*
Duration of diabetes (years)	3.5 ± 4.8	4.5 ± 6.6	4.6 ± 5.6
Height (cm)	157 ± 11	160 ± 13	166 ± 9*
Weight (kgs)	53.3 ± 12.5	49.4 ± 13.8	74.1 ± 15.2*
Body mass index (kg/m^2^)	21.5 ± 3.9	18.9 ± 3.8	26.8 ± 4.7*
Fasting plasma glucose (mg/dl)	193 ± 80	239 ± 110	172 ± 59*
Glycated Haemoglobin (%)	8.6 ± 2.1	11.1 ± 2.9	8.6 ± 1.8*
Total cholesterol (mg/dl)	173 ± 38	167 ± 39	179 ± 39*
Serum triglycerides (mg/dl)	113 ± 71	105 ± 82	177 ± 114*
HDL cholesterol (mg/dl)	42 ± 9	46 ± 12	38 ± 8*
LDL cholesterol (mg/dl)	109 ± 30	99 ± 31	106 ± 33*
Serum Creatinine (mg/dl)	0.6 ± 0.2	0.7 ± 0.4	0.7 ± 0.2
Fasting C- peptide (ng/ml)	1.5 ± 0.6	0.6 ± 0.3	3.0 ± 1.2*
Stimulated C- peptide (ng/ml)	3.9 ± 1.5	0.9 ± 0.3	6.9 ± 2.4*
Diabetes treatment n (%)			
Insulin	6 (17.6)	1010 (100)	–
OHA	14 (41.2)	–	1575 (98.1)
Insulin and OHA	14 (41.2)	–	29 (1.8)
Diet and Exercise	–	–	2 (0.1)
Other treatment n (%)			
Dyslipidemia	5 (14.7)	115 (11.4)	713 (44.4)
Hypertension	1 (2.9)	103 (10.2)	376 (23.4)

Data presented as mean ± standard deviation.

*Significant at *p* < 0.05 level using one way ANOVA, # Chi square comparison.

**+**The 34 MODY participants include HNF1α MODY, n = 21, HNF4 α MODY, n = 10 and ABCC8 MODY, n = 3.

**Figure 1:**
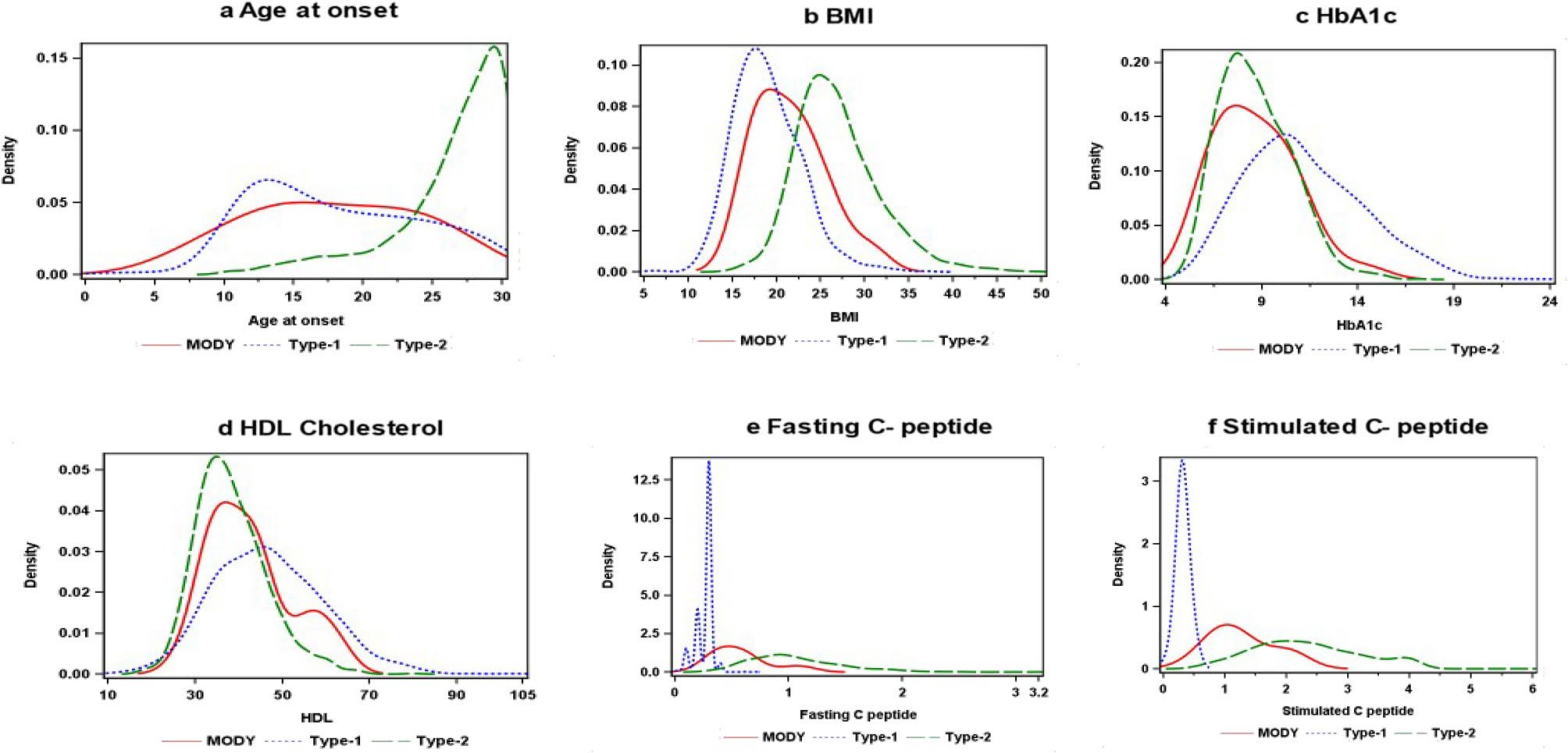
(**a**)–(**f**) Density plots for T1D, T2D and MODY.

Figure 1 presents the density plots for age at diagnosis of diabetes, BMI, HbA1c, HDL cholesterol values, and C-peptide (fasting and stimulated) values among MODY, T1D, and T2D. Even though there was some overlap in the distribution of age at diagnosis between the three groups, the age at diagnosis of T2D was clearly shifted to the right (Fig. 1a). Similarly, the BMI overlapped between the three groups, but MODY was clearly in between T1D and T2D (Fig. 1b). HbA1c and HDL cholesterol values showed considerable overlap between MODY, T1D, and T2D (Fig. 1c,d). Fasting C-peptide levels showed MODY to be in between T1D and T2D (Fig. 1e); however, with respect to stimulated C-peptide levels, the discrimination between the three groups was better, with T1D having the lowest stimulated C-peptide levels, followed by MODY while T2D had the highest values (Fig. 1f).

**Figure 2:**
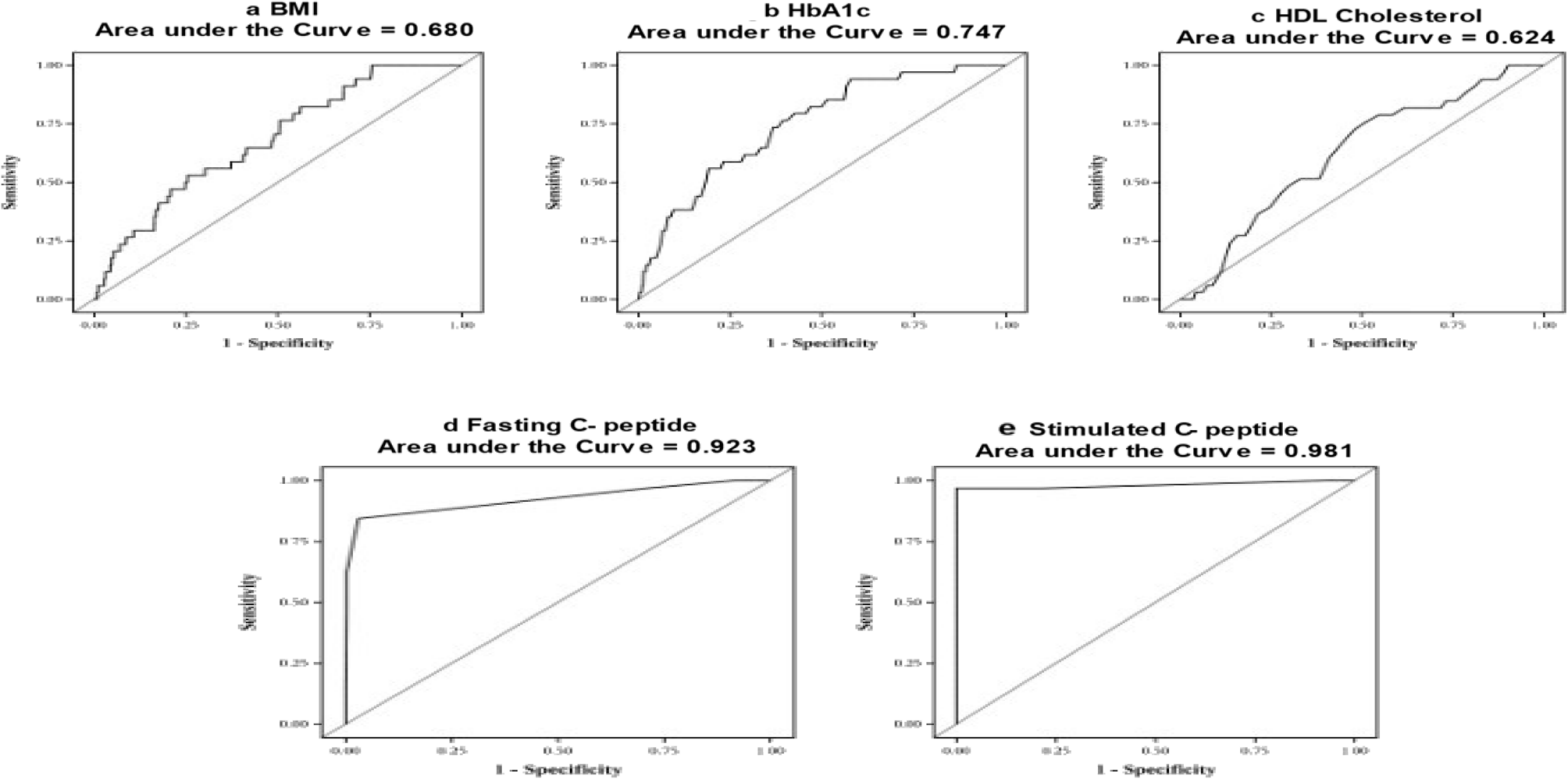
(**a**)–(**e**) ROC curves for T1D versus MODY.

**Figure 3:**
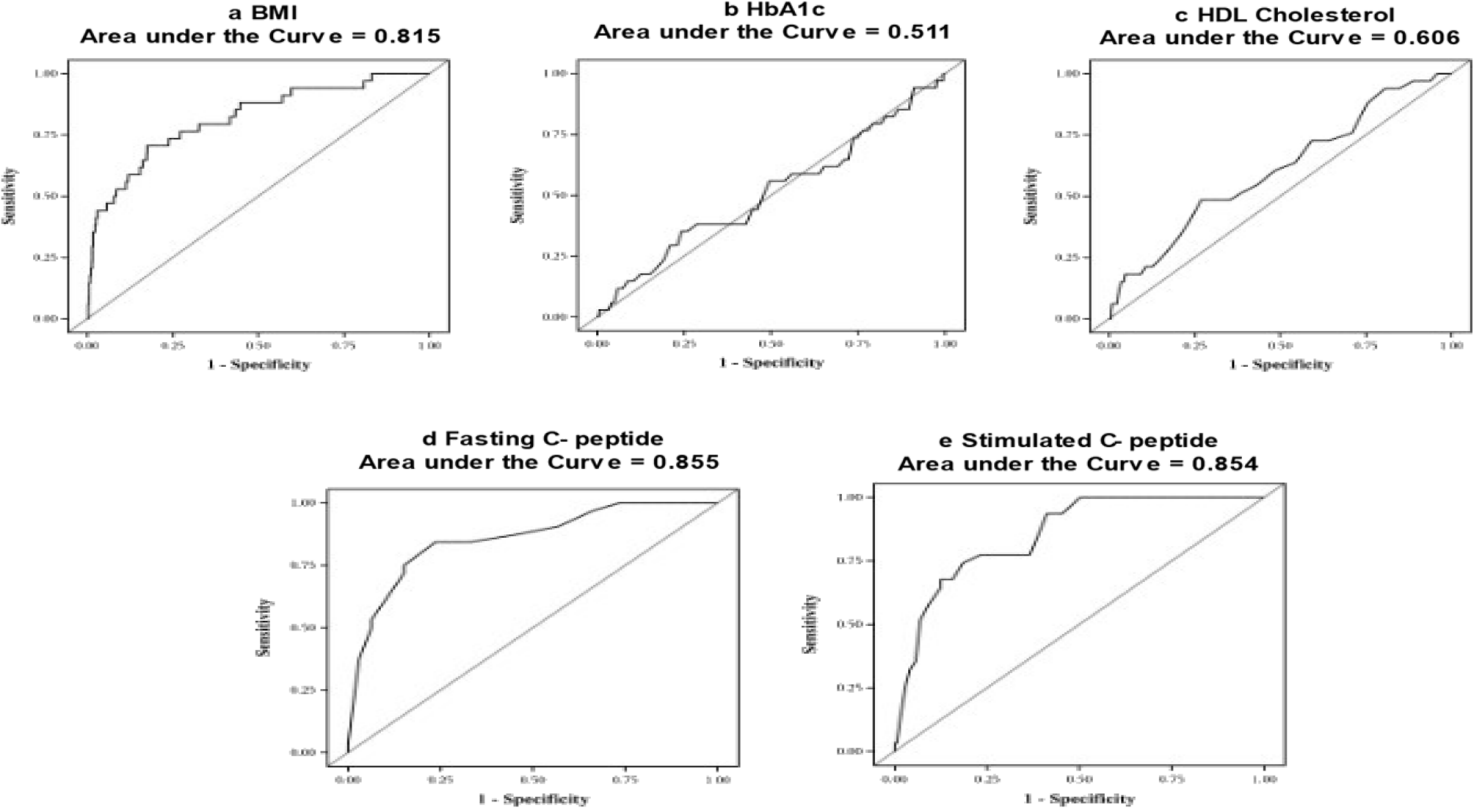
(**a**)–(**e**) ROC curves for T2D versus MODY.

ROC curves were constructed to identify whether these clinical parameters could help characterize cases to be referred for MODY genetic screening. ROCs are shown in Figs. 2 and 3. When MODY was compared with T1D (Figs. 2a–e), the fasting and stimulated C-peptide showed excellent discrimination. When MODY was compared to T2D (Figs. 3a–e), C-peptide (fasting and stimulated) and BMI showed good discrimination.

The sensitivity and specificity based on the ROC curves for the age of onset of diabetes, glycated hemoglobin, BMI, C-peptide (fasting and stimulated), and HDL cholesterol levels of MODY participants as compared with T1D and T2D, are presented in Tables 2 and 3. Sensitivity and specificity were considered to be suitable when both were above 70%. When MODY was compared to T1D (Table 2), the C-peptide (fasting and stimulated) levels showed the best sensitivity and specificity compared to the other clinical parameters. When MODY was compared to T2D, body mass index, and C-peptide (fasting and stimulated) levels showed top sensitivity and specificity.

**Table 2:** Cut off values for MODY (n = 34) when compared to T1D.

Variables	Cut-off point	Sensitivity	Specificity	PPV	NPV	AUC	Accuracy
BMI (kg/m^2^)	21.2	52.9	74.6	6.54	97.9	0.68	0.73
HbA1c (%)	10.0	76.5	60.6	6.13	98.7	0.75	0.61
HDL Cholesterol (mg/dl)	45	72.7	51.9	4.70	98.3	0.62	0.52
Fasting C- peptide (ng/ml)	1.2	84.4	96.9	46.55	99.5	0.92	0.96
Stimulated C- peptide (ng/ml)	2.1	96.8	100	100	99.9	0.98	0.99

*BMI* Body mass index, *HbA1c* Glycated hemoglobin, *HDL* High Density Lipopprotein.

**Table 3:** Cut off values for MODY (n = 34) when compared to T2D.

Variables	Cut-off point	Sensitivity	Specificity	PPV	NPV	AUC	Accuracy
BMI (kg/m^2^)	22.7	70.6	82.6	7.9	99.3	0.81	0.82
HbA1c (%)	7.2	35.3	75.6	2.9	98.2	0.51	0.74
HDL Cholesterol (mg/dl)	43	48.5	73.1	3.6	98.6	0.61	0.72
Fasting C- peptide (ng/ml)	2.1	84.4	76.3	6.6	99.6	0.85	0.76
Stimulated C- peptide (ng/ml)	4.5	74.2	81.4	7.2	99.4	0.85	0.81

*BMI* Body mass index, *HbA1c* Glycated hemoglobin, *HDL* High Density Lipopprotein.

### Identification of optimum cut-off points

To select optimum cut-off points, the shortest distance on the ROC curve was taken and by combining both T1D and T2D values, and the cut points for various clinical parameters were then derived for MODY. The identified probable parameters and their cut points to identify cases for MODY genetic testing were **a**. BMI 21.2–22.7 kg/m^2^, **b**. glycated hemoglobin 7.2–10%, **c**. HDL cholesterol 43–45 mg/dl, **d**. fasting C -peptide, 1.2–2.1 ng/ml and stimulated C -peptide, 2.1–4.5 ng/ml.

## Discussion

This study showed that MODY patients exhibit clinical and biochemical parameters (BMI, total cholesterol, triglycerides, HDL cholesterol, and C-peptide levels) that are intermediate between T1D and T2D. Although there was an overlap between the three diabetes subtypes, when ROC curves were constructed using the various clinical variables, fasting and stimulated C-peptide levels were found to be most useful to differentiate MODY from T1D, while C-peptide (fasting and stimulated, particularly the latter) and BMI were most useful to distinguish MODY from T2D. Also, optimal cut-off points to identify cases for MODY genetic testing were derived.

There are several obstacles in the identification of MODY patients for genetic screening. The reasons are largely due to the overlapping clinical features with T1D and T2D as only 50% of all MODY cases meet the traditional clinical criteria for MODY^33^. Other challenges include lack of a single criterion to diagnose MODY or T2D and the emphasis of most doctors in India on starting treatment straightaway rather than trying to establish a firm diagnosis^34^. Also, genetic testing is expensive although it is the gold standard for MODY diagnosis^35^. Hence identification of these cut points based on biochemical parameters could help in the Indian clinical setting to choose individuals to refer for MODY genetic testing to make it more cost effective. Studies across the world have shown pick-up rates for GCK-MODY to be between 12.5 and 61% and for HNF1A-MODY to be between 4.4 and 14% ^36–39^. In our recent study, we have shown a pick-up rate of MODY as 10.9% at our centre^40^ and this lower pick-up rate is likely because T2D presents at a lower age in India. This makes it even more important to identify biochemical parameters with good sensitivity in the Indian population to identify individuals to send for MODY genetic testing.

In this study, four clinical parameters, namely BMI, glycated hemoglobin, HDL and C-peptide (fasting and stimulated), were identified to compare the participants with MODY, T1D and T2D. The earliest MODY prediction model was developed in the United Kingdom (UK) led by Hattersley’s group in 2012 based on patients of white European origin. This model included the parameters: a. age at diagnosis, b. current age, c. BMI, d. glycated hemoglobin, e. parents with history of diabetes, f. sex and g. treatment with insulin or OHA. However, their age of onset of diabetes was less than 35 years, a criterion that our study suggests would not be useful in Asian Indian populations given the high proportion of T2D under this age^20^. Supporting the different findings in this Caucasian population with our Asian Indian population, the usage of this calculator in different populations have recently been studied with mixed results^41–47^.

Recently, a MODY prediction model was developed for a Chinese population which included a. major and b. specific standards^19^. The major criteria included age at diagnosis, positive antibody (ICA, GAD, IA2- Ab), BMI, family history of diabetes and fasting C-peptide and was used to assess the possibility of MODY with 7-point entry specificity criteria. Specific criteria are utilised for two MODY subtypes namely *HNF1A*-MODY3 and *GCK*-MODY2 where Sanger sequencing was recommended. The indicators identified for *GCK*-MODY2 were glycated hemoglobin, 2-h post prandial glucose and high sensitivity C-reactive protein (hs-CRP) while those for *HNF1A*-MODY3 included hs-CRP, triglycerides and others. This provides an advanced and more pragmatic approach to identify the right patients for genetic testing^19^. This model, developed for the Chinese population, is the one of the earliest developments for MODY diagnosis in an Asian population. The identification of the cut-off points for MODY patients in India also holds significance as China and India have the highest numbers of individuals with diabetes in the world. However, the Chinese model included participants of age less than 45 years again, a criterion not likely to be useful in our Asian Indian population further highlighting the importance of our study and the development of ethnic-specific calculators. It is also interesting to note that fasting C-peptide levels identified as one of the cut-off values in our model was also identified in the Chinese model, however this was not measured when developing the UK model. Therefore, fasting and stimulated C-peptide could be considered in future studies as it has the potential to be a significant variable while screening other populations.

As highlighted in both the UK and Chinese MODY calculators, BMI can be an important criterion for identification of MODY cases, with MODY patients usually having low BMI. However, recent studies have shown that MODY can also present with obesity which could pose an additional challenge to differentiate MODY from T2D^40^.This was also observed in our Asian Indian population, where the BMI of MODY patients overlapped with T1D and T2D. The TODAY study done among adolescents aged 10–17 years, reported that 4.5% of overweight/obese participants had monogenic diabetes and highlighted the difficulty of differentiating between the different youth-onset forms of diabetes^48^.

Other studies have reported higher HDL cholesterol levels among *HNF1A*-MODY3 patients and indicated that this could be useful to differentiate T2D and *HNF1A*-MODY3^19,49^. However, in our study, this was not observed, perhaps due to the small sample size or incorporation of all common MODY subtypes. A study from Poland reported that *HNF1A*-MODY3 participants had higher C-peptide values as compared to T1D, and this was suggested as a good discriminant^49^. Our study shows that this finding is also relevant in Indian populations, therefore this risk factor could be more broadly applicable to South Asian populations.

Among the clinical cut points, BMI is one of the useful ones. The identification of optimal cut off points for BMI (21.2–22.7 kg/m^2^), alongside the other biochemical criteria, could be a first step towards clinical screening for MODY patients in India. For example, whenever a young individual with diabetes comes for evaluation, if their BMI is less than 21.2 kg/m^2^, they might more likely have T1D, whereas if their BMI is above 22.7 kg/m^2^, they will more likely have T2D. Similarly, HbA1c is a useful parameter with a cut point of < 7.2% to ‘rule in’ or > 10% to ‘rule out’ MODY. It must be emphasized however that at the time of their first visit, in all young onset diabetes, the C peptide levels may be low due to glucotoxicity. As it can improve on follow up, a repeat C-peptide measurement could help in distinguishing or identifying the correct type of diabetes, after their glucotoxicity is corrected as we have shown earlier^30,31^.

The strengths of our study are first, that this is one of the first from south Asia to identify clinical and biochemical parameters for genetic screening for MODY. Secondly, the parameters used are routinely done in a clinic setting to classify young onset diabetes and thirdly accurate genotyping of MODY was done using the ACMG and GnomAD criteria.

There are a few limitations of our study. Firstly, we have only included a small number of MODY participants. However, given that these were identified from a large cohort of patients referred to a single centre, a large multi-centre study would be required to get larger numbers of MODY. Secondly, after genetic screening, the remaining 496 persons were not included. Although they are most likely to be T2D, there is a possibility that they may harbour some unknown or novel MODY genes for which whole genome or exome sequencing is needed, which could not be done.

In summary, we report on the development of cut points for selecting the most appropriate candidates for MODY genetic testing among youth onset diabetes in India. In a diabetes clinic set up, where young diabetes patients are registered, serial follow up of biochemical parameters, especially C peptide assay and prompt referral for MODY genetic screening will ensure more accurate diagnosis and hence prevent unnecessary insulin usage besides improving their quality of life. We believe that the findings of this study will be the first step towards development of a MODY calculator for India.

## Data Availability

The datasets generated and analysed during the current study are not publicly available due to the inclusion of patient’s information but are available from the corresponding author on reasonable request.

## References

[1] International Diabetes Federation Atlas. Brussels, Belgium: International Diabetes Federation (2021).

[2] Tandon N, Anjana RM, Mohan V, Kaur T, Afshin A, Ong K (2018). The increasing burden of diabetes and variations among the states of India: The Global Burden of Disease Study 1990–2016. Lancet Glob. Health.

[3] Praveen PA, Madhu SV, Mohan V, Das S, Kakati S, Shah N (2016). Registry of Youth Onset Diabetes in India (YDR): Rationale, recruitment, and current status. J. Diabet. Sci. Technol..

[4] Sahoo SK, Zaidi G, Vipin VP, Chapla A, Thomas N, Yu L (2019). Heterogeneity in the aetiology of diabetes mellitus in young adults: A prospective study from north India. Indian J. Med. Res..

[5] Praveen PA, Madhu SV, Mohan V, Das S, Kakati S, Shah N (2016). Registry of Youth Onset Diabetes in India (YDR): Rationale, recruitment, and current status. J Diabet. Sci. Technol..

[6] Mohan V, Ramachandran A, Snehalatha C, Mohan R, Bharani G, Viswanathan M (1985). High prevalence of maturity-onset diabetes of the young (MODY) among Indians. Diabet. Care.

[7] Anuradha S, Radha V, Deepa R, Hansen T, Carstensen B, Pedersen O (2005). A prevalent amino acid polymorphism at codon 98 (Ala98Val) of the hepatocyte nuclear factor-1α is associated with maturity-onset diabetes of the young and younger age at onset of type 2 diabetes in Asian Indians. Diabet. Care.

[8] Radha V, Ek J, Anuradha S, Hansen T, Pedersen O, Mohan V (2009). Identification of novel variants in the hepatocyte nuclear factor-1α gene in South Indian patients with maturity onset diabetes of young. J. Clin. Endocrinol. Metab..

[9] Anuradha S, Radha V, Mohan V (2011). Association of novel variants in the hepatocyte nuclear factor 4A gene with maturity onset diabetes of the young and early onset type 2 diabetes. Clin. Genet..

[10] Kanthimathi S, Jahnavi S, Balamurugan K, Ranjani H, Sonya J, Goswami S (2014). Glucokinase gene mutations (MODY 2) in Asian Indians. Diabet. Technol. Ther..

[11] Kanthimathi S, Balamurugan K, Mohan V, Shanthirani CS, Gayathri V, Radha V (2015). Identification and molecular characterization of HNF1B gene mutations in Indian diabetic patients with renal abnormalities. Ann. Hum. Genet..

[12] Colclough K, Patel K (2022). How do i diagnose maturity onset diabetes of the young in my patients?. Clin. Endocrinol..

[13] Tosur M, Philipson LH (2022). Precision diabetes: Lessons learned from maturity-onset diabetes of the young (MODY). J Diabet. Investig..

[14] Kant R, Davis A, Verma V (2022). Maturity-onset diabetes of the young: Rapid evidence review. Am Fam Physician..

[15] Sampathkumar G, Valiyaparambil PP, Kumar H, Bhavani N, Nair V, Menon U (2021). Low genetic confirmation rate in South Indian subjects with a clinical diagnosis of maturity-onset diabetes of the young (MODY) who underwent targeted next-generation sequencing for 13 genes. J. Endocrinol. Investig..

[16] Unnikrishnan R, Radha V, Mohan V (2021). Challenges involved in incorporating personalised treatment plan as routine care of patients with diabetes. Pharmgenomics Pers. Med..

[17] Shields BM, Hicks S, Shepherd MH, Colclough K, Hattersley AT, Ellard S (2010). Maturity-onset diabetes of the young (MODY): How many cases are we missing?. Diabetologia.

[18] Firdous P, Nissar K, Masoodi SR, Ganai BA (2022). Biomarkers: Tools for discriminating MODY from other diabetic subtypes. Indian J. Endocrinol. Metab..

[19] Fu J, Ping F, Wang T, Liu Y, Wang X, Yu J (2021). A clinical prediction model to distinguish maturity-onset diabetes of the young from type 1 and type 2 diabetes in the Chinese population. Endocr. Pract..

[20] Shields BM, McDonald TJ, Ellard S, Campbell MJ, Hyde C, Hattersley AT (2012). The development and validation of a clinical prediction model to determine the probability of MODY in patients with young-onset diabetes. Diabetologia.

[21] Misra S, Shields B, Colclough K, Johnston DG, Oliver NS, Ellard S (2016). South Asian individuals with diabetes who are referred for MODY testing in the UK have a lower mutation pick-up rate than white European people. Diabetologia.

[22] Tattersall RB, Fajans SS (1975). A difference between the inheritance of classical juvenile onset and maturity onset type diabetes of young people. Diabetes.

[23] Karczewski KJ, Francioli LC, Tiao G, Cummings BB, Alföldi J, Wang Q (2020). The mutational constraint spectrum quantified from variation in 141,456 humans. Nature.

[24] Richards S, Aziz N, Bale S, Bick D, Das S, Gastier-Foster J (2015). Standards and guidelines for the interpretation of sequence variants: a joint consensus recommendation of the American College of Medical Genetics and Genomics and the Association for Molecular Pathology. Genet. Med. Offic. J. Am. Coll. Med. Genet..

[25] Vasileiou G, Hoyer J, Thiel CT, Schaefer J, Zapke M, Krumbiegel M (2019). Prenatal diagnosis of HNF1B-associated renal cysts: Is there a need to differentiate intragenic variants from 17q12 microdeletion syndrome?. Prenat Diagn..

[26] Taghavi SM, Fatemi SS, Rafatpanah H, Ganjali R, Tavakolafshari J, Valizadeh N (2009). Mutations in the coding regions of the hepatocyte nuclear factor 4 alpha in Iranian families with maturity onset diabetes of the young. Cardiovasc. Diabetol..

[27] Tinto N, Zagari A, Capuano M, De Simone A, Capobianco V, Daniele G (2008). Glucokinase gene mutations: Structural and genotype-phenotype analyses in MODY children from South Italy. PLoS ONE.

[28] Niu X, Perakakis N, Laubner K, Limbert C, Stahl T, Brendel MD (2007). Human Krüppel-like factor 11 inhibits human proinsulin promoter activity in pancreatic beta cells. Diabetologia.

[29] Ellard S, Flanagan SE, Girard CA, Patch AM, Harries LW, Parrish A (2007). Permanent neonatal diabetes caused by dominant, recessive, or compound heterozygous SUR1 mutations with opposite functional effects. Am. J. Hum. Genet..

[30] Amutha A, Datta M, Unnikrishnan R, Anjana RM, Mohan V (2012). Clinical profile and complications of childhood- and adolescent-onset type 2 diabetes seen at a diabetes Center in South India. Diabet. Technol. Ther..

[31] Snehalatha C, Ramachandran A, Mohan V, Viswanathan M (1987). Pancreatic beta cell response in insulin treated NIDDM patients limitations of a random C-peptide measurement. Diabet. Metab..

[32] Alberti KG, Zimmet PZ (1998). Definition, diagnosis and classification of diabetes mellitus and its complications: Part 1: Diagnosis and classification of diabetes mellitus provisional report of a WHO consultation. Diabet. Med..

[33] Urbanová J, Brunerová L, Brož J (2018). Hidden MODY-Looking for a Needle in a Haystack. Front. Endocrinol..

[34] Peixoto-Barbosa R, Reis AF, Giuffrida FMA (2020). Update on clinical screening of maturity-onset diabetes of the young (MODY). Diabetol. Metab. Syndr..

[35] Amed S, Oram R (2016). Maturity-onset diabetes of the young (MODY): Making the right diagnosis to optimize treatment. Can. J. Diabet..

[36] Hattersley AT (1998). Maturity-onset diabetes of the young: Clinical heterogeneity explained by genetic heterogeneity. Diabet. Med..

[37] Mantovani V, Salardi S, Cerreta V, Bastia D, Cenci M, Ragni L (2003). Identification of eight novel glucokinase mutations in Italian children with maturity-onset diabetes of the young. Hum. Mutat..

[38] Pihoker C, Gilliam LK, Ellard S, Dabelea D, Davis C, Dolan LM (2013). Prevalence, characteristics and clinical diagnosis of maturity onset diabetes of the young due to mutations in HNF1A, HNF4A, and glucokinase: Results from the SEARCH for Diabetes in Youth. J. Clin. Endocrinol. Metab..

[39] Gragnoli C, Cockburn BN, Chiaramonte F, Gorini A, Marietti G, Marozzi G (2001). Early-onset Type II diabetes mellitus in Italian families due to mutations in the genes encoding hepatic nuclear factor 1α and glucokinase. Diabetologia.

[40] Aarthy R, Aston-Mourney K, Mikocka-Walus A, Radha V, Amutha A, Anjana RM (2021). Clinical features, complications and treatment of rarer forms of maturity-onset diabetes of the young (MODY): A review. J. Diabet. Complicat..

[41] Junior, A., Magalhães, Á., Tedesco, F., Santana, L., Franco, P., Freitas, S., *et al*. The performance of the MODY calculator in a non-Caucasian, mixed-race population diagnosed with diabetes mellitus before age 35 years2022. 10.1186/s13098-023-00985-3 PMC9900997 36747290

[42] da Silva ST, Fonseca L, Santos Monteiro S, Borges Duarte D, Martins Lopes A, Couto de Carvalho A (2022). MODY probability calculator utility in individuals' selection for genetic testing: Its accuracy and performance. Endocrinol. Diabet. Metab..

[43] Szopa M, Klupa T, Kapusta M, Matejko B, Ucieklak D, Glodzik W (2019). A decision algorithm to identify patients with high probability of monogenic diabetes due to HNF1A mutations. Endocrine.

[44] Hohendorff J, Zapala B, Ludwig-Slomczynska AH, Solecka I, Ucieklak D, Matejko B (2019). The utility of MODY probability calculator in probands of families with early-onset autosomal dominant diabetes from Poland. Minerva Med..

[45] Ang SF, Lim SC, Tan C, Fong JC, Kon WY, Lian JX (2016). A preliminary study to evaluate the strategy of combining clinical criteria and next generation sequencing (NGS) for the identification of monogenic diabetes among multi-ethnic Asians. Diabetes Res. Clin. Pract..

[46] Tarantino RM, Abreu GM, Fonseca ACP, Kupfer R, Pereira MFC, Campos Júnior M (2020). MODY probability calculator for GCK and HNF1A screening in a multiethnic background population. Arch. Endocrinol. Metab..

[47] Kwak SH, Jung CH, Ahn CH, Park J, Chae J, Jung HS (2016). Clinical whole exome sequencing in early onset diabetes patients. Diabet. Res Clin Pract..

[48] Kleinberger JW, Copeland KC, Gandica RG, Haymond MW, Levitsky LL, Linder B (2017). Monogenic diabetes in overweight and obese youth diagnosed with type 2 diabetes: The TODAY clinical trial. Genet. Med..

[49] Fendler W, Borowiec M, Antosik K, Szadkowska A, Deja G, Jarosz-Chobot P (2011). HDL cholesterol as a diagnostic tool for clinical differentiation of GCK-MODY from HNF1A-MODY and type 1 diabetes in children and young adults. Clin. Endocrinol..

